# Predictors and risk model for positive circumferential resection margin after robot-assisted total mesorectal excision: retrospective cohort study

**DOI:** 10.1093/bjsopen/zraf027

**Published:** 2025-05-22

**Authors:** Ritch T J Geitenbeek, Thijs A Burghgraef, Rauand Duhoky, Christina A Fleming, Aurore Moussion, Nabila Bouazza, Eddy Cotte, Anne Dubois, Eric Rullier, Quentin Denost, Philippe Rouanet, Jim Khan, Roel Hompes, Esther C J Consten, the EUREKA study group, G J D van Acker, T S Aukema, H J Belgers, F H Beverdam, J G Bloemen, K Bosscha, S O Breukink, P P L O Coene, R M P H Crolla, P van Duijvendijk, E B van Duyn, I F Faneyte, S A F Fransen, A A W van Geloven, M F Gerhards, W M U van Grevenstein, K Havenga, I H J T de Hingh, C Hoff, G Kats, J W A Leijtens, M F Lutke Holzik, J Melenhorst, M M Poelman, A Pronk, A H W Schiphorst, J M J Schreinemakers, C Sietses, A B Smits, I Somers, E J Spillenaar Bilgen, H B A C Stockmann, A K Talsma, P J Tanis, J Tuynman, E G G Verdaasdonk, F A R M Warmerdam, H L van Westreenen, D D E Zimmerman, Quentin Denost, Christina Fleming, Clinique Tivoli, Philipe Rouanet, Nabila Bouazza, Aurore Moussion, Eddy Cotte, Anne Dubois, Chu Estaing, Eric Rullier, J Conti, G David, D O'Leary, A Przedlacka, M Rabie, J Richardson, F Sagias, P Sykes

**Affiliations:** Department of Surgery, University of Groningen, University Medical Centre Groningen, Groningen, The Netherlands; Department of Surgery, Meander Medical Centre, Amersfoort, The Netherlands; Department of Surgery, University of Groningen, University Medical Centre Groningen, Groningen, The Netherlands; Department of Surgery, Meander Medical Centre, Amersfoort, The Netherlands; Department of Colorectal Surgery, Portsmouth Hospitals University NHS Trust and the University of Portsmouth, Portsmouth, UK; Bordeaux Colorectal Institute, Clinique Tivoli, Bordeaux, France; Clinical Research Department, Montpellier Cancer Institute (ICM), University of Montpellier, Montpellier, France; Clinical Research Department, Montpellier Cancer Institute (ICM), University of Montpellier, Montpellier, France; Department of Digestive and Oncological Surgery, Lyon University Hospital, Lyon-Sud Hospital, Pierre-Bénite, France; Department of Colorectal Surgery, Chu Estaing, Clermont-Ferrand, France; Department of Digestive Surgery, Colorectal Unit, Haut-Lévêque Hospital, Bordeaux University Hospital, Pessac, France; Bordeaux Colorectal Institute, Clinique Tivoli, Bordeaux, France; Surgery Department, Montpellier Cancer Institute (ICM), University of Montpellier, Montpellier, France; Department of Colorectal Surgery, Portsmouth Hospitals University NHS Trust and the University of Portsmouth, Portsmouth, UK; Department of Surgery, University of Amsterdam, University Medical Centre Amsterdam, Amsterdam, The Netherlands; Department of Surgery, Amsterdam Cancer Centre, Amsterdam, The Netherlands; Department of Surgery, University of Groningen, University Medical Centre Groningen, Groningen, The Netherlands; Department of Surgery, Meander Medical Centre, Amersfoort, The Netherlands

## Abstract

**Background:**

Positive circumferential resection margin (CRM) after total mesorectal excision (TME) is associated with higher local and systemic recurrence rates, affecting overall survival in patients with rectal cancer. Although risk factors for positive CRM have been identified for open, laparoscopic, and transanal TME, these may differ for robot-assisted total mesorectal excision (R-TME). This study aimed to assess the incidence of positive CRM following R-TME and identify the associated preoperative risk factors.

**Method:**

An international multicentre retrospective study included patients receiving R-TME between January 2013 and January 2022 in centres based in the Netherlands, UK, and France. Endpoints were the incidence of and predictive factors for positive CRM. Univariable and multivariable logistic regression analyses were performed, and factors associated with positive CRM were then assessed by formulating a predictive model.

**Results:**

A total of 1390 patients underwent R-TME, and the incidence of positive CRM was 6.0% (*n* = 83). Multivariable analysis revealed significant associations between positive CRM and cT4 tumours (OR 2.27), involved mesorectal fascia on staging magnetic resonance imaging at diagnosis (OR 1.89), and non-sphincter-saving surgery (OR 2.22). The predictive model exhibited satisfactory discrimination (area under the receiver-operating characteristic curve > 0.7) and predicted a 26% risk of positive CRM when all identified risk factors were present.

**Conclusion:**

Preoperative tumour- and procedure-related factors, rather than patient-related factors, are associated with CRM involvement after R-TME. The proposed predictive model allows preoperative calculation of the risk of positive CRM, offering valuable insights for optimizing patient selection and tailoring treatment approaches to enhance oncological outcomes.

## Teaser

This study assessed the incidence of positive circumferential resection margin (CRM) following robot-assisted total mesorectal excision (R-TME) and identified associated preoperative risk factors. Among 1390 patients, a 6.0% positive CRM rate was observed, with cT4 tumours, involved mesorectal fascia, and non-sphincter-saving surgery being significant predictors.

## Introduction

Total mesorectal excision (TME), initially introduced by Heald and Ryall in 1982, has since evolved into the standard surgical treatment for operable rectal cancer^1^. Over the past decades, further advancements, such as the integration of preoperative staging modalities and neoadjuvant therapy, have collectively improved the clinical outcomes and overall survival for patients with rectal cancer^2^. More recently, robot-assisted TME (R-TME) has emerged, which may enhance surgical precision and oncological outcomes, particularly in low rectal cancers^3^.

The critical objective in TME surgery is to achieve tumour clearance with a clear circumferential resection margin (CRM), as this parameter is the most important short-term predictor of both local and systemic recurrence^4,5^. Predictive factors for obtaining a positive CRM have been extensively investigated for conventional laparoscopic TME (L-TME), revealing the influence of various tumour- and patient-related factors. Tumour characteristics such as anterior location and cT4 status, as well as patient-related variables like male gender and obesity, have been established as risk factors that increase the technical complexity of open and conventional L-TME and increase the likelihood of a positive CRM^6–8^. Recent research examining predictive factors in patients undergoing transanal TME (TaTME) revealed a striking disparity, with only tumour-related characteristics demonstrating predictive value, whereas patient-related factors, known to impact CRM rates in L-TME, exhibited no predictive value^9^. The literature lacks studies elucidating predictive factors for positive CRM following R-TME. The difference between L-TME and TaTME underscores the possibility that predictive factors for R-TME may differ substantially from those of conventional laparoscopic approaches as well.

To address this knowledge gap, the present study aimed to determine the incidence of positive CRM after R-TME procedures performed by experienced surgeons within an international R-TME collaborative. In addition, the aim was to construct a predictive model that identifies preoperative factors serving as reliable indicators for positive CRM in the context of R-TME procedures.

## Methods

### Patient selection

An international multicentre retrospective data set was used for analysis. The data originated from high-volume robotic rectal cancer centres in France, the UK, and the Netherlands; the Expert Dutch, French, and UK robotic rectal cancer centres (EUREKA) collaborative^10^. The data set included patients receiving R-TME between January 2013 and January 2022 by surgeons who had surpassed their learning curve. The learning curve for R-TME was defined as having performed over 50 procedures using this specific technique. This data set included only adult patients over the age of 18 with biopsy-confirmed rectal cancer located within 15 cm from the anorectal junction (ARJ) on magnetic resonance imaging (MRI), receiving curative and elective R-TME. The current study excluded complete responders to neoadjuvant therapy and patients whose CRM status was unavailable. The institutional review board of each participating centre granted ethical approval for the EUREKA collaborative. Informed consent was deemed unnecessary by the Medical Ethical Committee as patients were pseudonymized, and pseudonymization keys remained at their respective sites.

### Data extraction and outcome parameters

The EUREKA data set^10^ is a comprehensive collection of retrospective patient data encompassing patient demographics, tumour staging and neoadjuvant treatment (NAT), operative specifics, postoperative clinical and histological outcomes, readmission details, late morbidity, and long-term oncologic follow-up. The primary focus of this study was two-fold: to determine the incidence of positive CRM and identify possible predictive factors. Positive CRM was defined as a tumour within 1 mm from the CRM. Additional definitions of extracted data and outcome parameters are provided in *File S1*. The data reporting adhered to the STROBE criteria for observational cohort studies^11^. In instances of missing data or potentially erroneous results, participating parties were individually contacted via email up to three times within one month to seek clarification or adjustments.

### Statistical analysis

The statistical analyses were conducted using R version 4.3.0 (R Core Team, 2023). Missing data for variables within the model were handled through multiple imputations with predictive mean, assuming a (completely) random pattern. No instances of non-random missing data were observed. For other variables not included in the predictive model, missing values did not exceed 15%. The presented percentages reflect the available data after excluding missing values. Categorical variables were expressed as absolute numbers and percentages. Continuous data was presented as mean values and standard deviations or median and interquartile range, depending on the distribution. Continuous variables, such as body mass index (BMI) and tumour height relative to the ARJ, were stratified into clinically relevant subgroups. Intergroup comparisons were performed using the chi-square test for categorical variables and the Mann–Whitney U test for continuous variables. The selection of variables for inclusion in the multivariable analysis was based on well-established a-priori risk factors for positive CRM, as documented in the current literature^7,8,12–17^. The following variables were evaluated as potential predictors for a positive CRM: male sex, BMI, prior abdominal surgery, distance to ARJ, anterior tumour location, cT4 stage, ≥cN1 stage, extramural venous invasion on magnetic resonance imaging (mrEMVI), presence of involved mesorectal fascia (MRF) on MRI, sphincter-saving surgery, and response to neoadjuvant therapy. Notably, the T-stage, N-stage, and MRF status reflect the status after neoadjuvant therapy if a patient received neoadjuvant therapy. Variables that exhibited a significance level of *P* < 0.05 using backward step selection in the multivariate regression analysis, or those with 95% confidence intervals not encompassing 1, were deemed significant and included as predictive factors in the CRM prediction model. The coefficients derived from the multivariate analysis were multiplied by 10 and used to weigh the predicted risk of positive CRM after R-TME for rectal cancer.

### Model validation

Although no external validation was performed, we internally validated the model by bootstrapping a random sample of 150 patients from the original study population. This method was deemed most suitable for this sample size^18,19^. Calibration, often called goodness-of-fit, assesses the model’s capability to assign probabilities of outcomes to individual patients accurately. To evaluate this, a plot was generated comparing the observed number of positive CRM cases to the expected number of positive CRM cases. Model discrimination (the ability to assign higher probabilities of CRM involvement to patients who develop an involved margin) was measured by area under the receiver-operating characteristic (ROC) curve, along with associated 95% confidence intervals.

## Results

All patients enrolled in the EUREKA collaborative data set from January 2013 to January 2022 were reviewed (*n* = 1622; *Fig. S1*). After eligibility criteria were applied, 1390 patients who had an R-TME met our inclusion criteria. The exclusions were attributed to a complete pathological response (pT0) following neoadjuvant therapy and unavailable CRM status.

*Table 1* provides a comprehensive overview of patient demographics, tumour characteristics, and pathological outcomes before data imputation. Among the cohort, 911 patients (65.5%) were male, with a mean(s.d.) BMI of 26.33(4.51) kg/m^2^. Notably, 380 patients (27.5%) had a history of prior abdominal surgery. The median distance from the tumour to the ARJ was 5 cm (i.q.r. 2.1–8.0), with 173 tumours (12.4%) positioned within 1 cm of the ARJ. Anterior tumour localization was observed in 190 (14.9%) patients. Preoperative staging indicated cT4 in 109 (8.0%) patients and ≥cN1 in 602 (52.6%). Overall, mrEMVI was identified in 504 (54.5%), and 576 patients (43.7%) exhibited a threatened MRF on baseline MRI. Chemoradiation was administered to 268 patients (19.3%), and 235 (16.9%) received total neoadjuvant therapy. Among those who underwent neoadjuvant therapy for tumour downsizing, 495 (86.8%) exhibited a favourable ‘response’ according to the tumour regression grading (TRG) on MRI, whereas 75 (13.2%) did not respond to the treatment. After receiving neoadjuvant therapy, 372 (26.8%) exhibited a threatened MRF on MRI. Sphincter-saving surgery was successfully performed in 1034 patients (76.6%). TME specimen quality was defined as complete or near complete in 1287 (94.6%) patients. A positive CRM was observed in 83 (6.0%) patients.

**Table 1:** Patient and tumour characteristics and pathological outcomes

Factor	EUREKA data results (*n* = 1390)
**Patient characteristics**	
Age (years), mean(s.d.)	67.49(11.46)
**Sex**	
Male	911 (65.5)
BMI, kg/m^2^, mean(s.d.)	26.33(4.51)
Overweight, BMI >30	245 (17.6)
ASA classification	
I	237 (17.1)
II	854 (61.4)
III	292 (21.0)
IV	7 (0.5)
Previous abdominal surgery	380 (27.5)
**Tumour characteristics**	
Distance to ARJ (cm), median (i.q.r.)	5.00 (2.10–8.00)
≤1 cm	173 (12.4)
Anterior tumour	190 (14.9)
Clinical T-stage	
cT1	29 (2.1)
cT2	398 (29.1)
cT3	833 (60.8)
cT4	109 (8.0)
Clinical N-stage	
cN0	542 (47.4)
cN1	430 (37.6)
cN2	172 (15.0)
Clinical M-stage	
cM0	1218 (92.3)
cM1	101 (7.7)
EMVI positive on baseline MRI	504 (54.5)
Involved MRF on baseline MRI	576 (43.7)
Neoadjuvant treatment	
None	592 (42.6)
Chemotherapy only	34 (2.4)
Short preoperative	227 (16.3)
Chemoradiation	268 (19.3)
Total neoadjuvant therapy (chemoradiation + chemo)	235 (16.9)
Other	33 (2.4)
Tumour response after neoadjuvant therapy	
Response	495/570 (86.8)
No response	75/570 (13.2)
Procedure type	
Restorative LAR	850 (63.0)
Non-restorative LAR (ultra-low Hartmann)	184 (13.6)
APR	316 (23.4)
**Pathological outcomes**	
Pathological T-stage	
pT1	124 (9.1)
pT2	502 (36.9)
pT3	702 (51.6)
pT4	33 (2.4)
Pathological N-stage	
pN0	853 (62.2)
pN1	412 (30.1)
pN2	106 (7.7)
Pathological M-stage	
pM0	835 (92.9)
pM1	64 (7.1)
Number of lymph nodes harvested, mean(s.d.)	17.61(9.00)
Histological type	
Adenocarcinoma	1074 (89.9)
Mucinous carcinoma	120 (10.1)
TME completeness	
Incomplete	73 (5.4)
Partially complete	180 (13.2)
Complete	1107 (81.4)
DRM (mm), median (i.q.r.)	10.00 (10.00–25.00)
CRM involvement	83 (6.0)

Values are *n* (%) unless otherwise indicated. APR, abdominal perineal resection; ARJ, anorectal junction; BMI, body mass index; CRM, circumferential resection margin; DRM, distal resection margin; EMVI, extramural venous invasion; LAR, low anterior resection; MRF, mesorectal fascia; TME, total mesorectal excision.

### Development of the predictive risk model

The assessed risk factors for CRM positivity after multiple imputation are reported in *Table 2* according to the CRM involvement rate, with event rates and multivariable analysis of key CRM involvement predictors reported in *Table 3*.

**Table 2:** The proportion of rectal cancer cases with CRM involvement according to assessed risk factors for CRM positivity

Factor	CRM negative (*n* = 1307)	CRM positive (*n* = 83)	*P*
**Patient characteristics**			
Sex			
Male	852 (65.2)	59 (71.1)	0.329
BMI, kg/m^2^, mean(s.d.)	26.38(4.50)	25.50(4.58)	0.084
Overweight >30	232 (17.8)	14 (16.9)	0.955
Previous abdominal surgery	353 (27.0)	29 (34.9)	0.149
**Tumour characteristics**			
Distance to ARJ (cm), median (i.q.r.)	5.00 (2.65–8.00)	3.00 (0.60–6.80)	<0.001
≤1 cm	151 (11.6)	22 (26.5)	<0.001
Anterior tumour	202 (15.5)	14 (16.9)	0.851
Clinical T-stage			
cT 4	67 (5.1)	16 (19.3)	<0.001
Clinical N-stage			
cN+	387 (29.6)	39 (47.0)	<0.001
EMVI positive on baseline MRI	725 (55.5)	57 (68.7)	0.025
Involved MRF on baseline MRI	431 (33.0)	49 (59.0)	<0.001
Tumour response after neoadjuvant therapy			
No neoadjuvant	548 (41.9)	32 (38.6)	
Response	664 (50.8)	40 (48.2)	
No response	95 (7.3)	11 (13.3)	<0.136
Procedure type			
Sphincter-saving	1025 (78.4)	49 (59.0)	
APR	282 (21.6)	34 (41.0)	<0.001

Values are *n* (%) unless otherwise indicated. APR, abdominal perineal resection; ARJ, anorectal junction; BMI, body mass index; CRM, circumferential resection margin; EMVI, extramural venous invasion; MRF, mesorectal fascia.

**Table 3:** Multivariable analysis of preoperative risk factors for CRM positivity in all patients (*n* = 1390)

Factor	Event rate (%)	Multivariate analysis		
		Odds ratio	95% c.i.	*P*
**Sex**				
Male				
Yes	59/911 (6.5%)	–	–	–
No	24/479 (5.0%)	Ref		
**BMI >30**				
Yes	14/246 (5.7%)	–	–	–
No	69/1144 (6.0%)	Ref		
**Previous abdominal surgery**				
Yes	29/382 (7.6%)	–	–	–
No	54/1008 (5.4%)	Ref		
**Distance from ARJ <1 cm**				
Yes	22/173 (12.7%)	–	–	–
No	61/1217 (5.0%)	Ref		
**Anterior tumour**				
Yes	14/216 (6.5%)	–	–	–
No	69/1174 (5.9%)	Ref		
**cT4 tumour**				
Yes	14/73 (19.2%)	2.27	1.106,4.466	0.020
No	69/1317 (5.2%)	Ref		
**cN ≥1**				
Yes	36/428 (8.4%)	–	–	–
No	47/962 (4.9%)	Ref		
**EMVI on MRI**				
Yes	59/773 (7.6%)	–	–	–
No	24/617 (3.9%)	Ref		
**Involved MRF on baseline MRI**				
Yes	49/480 (10.2%)	1.89	1.128,3.160	0.015
No	34/910 (3.7%)	Ref		
**Sphincter-saving surgery**				
Yes	49/1074 (4.6%)	Ref		
No	34/316 (10.8%)	2.22	1.377,3.540	0.001
**Neoadjuvant therapy response**				
No neoadjuvant	19/570 (3.3%)	–	–	–
Response	51/716 (7.1%)	Ref		
No response	13/104 (12.5%)	1.96	0.967,3.733	0.050

ARJ, anorectal junction; BMI, body mass index; CRM, circumferential resection margin; EMVI, extramural venous invasion; MRF, mesorectal fascia; Ref, reference.

Positive CRM was independently associated with cT4 tumours (OR 2.27, 95% c.i. 1.1 to 4.5), tumours with MRF involvement on MRI (OR 1.89, 95% c.i. 1.1 to 3.2), and patients receiving non-sphincter-saving surgery (APR) (OR 2.22, 95% c.i. 1.4 to 3.5). Exhibiting no response to neoadjuvant therapy was not considered a significant predictor, as the 95% c.i. of this potential predictor included 1. None of the patient-related variables (gender, BMI, or history of prior abdominal surgery) showed significant association with a positive CRM.

The individual risk factors' weights, denoting the natural logarithm of the respective odds ratios, are shown in *Table 4*. Notably, cT4 tumours and non-sphincter-saving surgery exhibited the highest weight (2.1). The weight assigned to MRF involvement on MRI risk factors is 1.8.

**Table 4:** Preoperative risk scoring for a positive CRM after R-TME based on prediction model

Risk factor	Weight
cT4 tumour	2.1
Involved MRF on baseline MRI	1.8
Non-sphincter-saving	2.1
Cumulative points	6.0

The coefficients derived from the multivariate analysis were multiplied by 10 and used as weights in the nomogram for predicting the risk of positive CRM after R-TME for rectal cancer. CRM, circumferential resection margin; MRF, mesorectal fascia; MRI, magnetic resonance imaging; R-TME, robot-assisted total mesorectal excision.

The ROC curve yielded an area under the curve (AUC) value of 0.716 (95% CI 0.660 to 0.716), and subsequent correction for optimism revealed a c-statistic of 0.712 (*Fig. S2*). Forest plots and gradient boost models allowed for including a larger number of predictors in the model, which slightly improved AUC values (>0.73).

*Figure S3* graphically illustrates the model-predicted risk of a positive CRM juxtaposed against the empirically observed incidence of positive CRM. *Table 5* provides the probabilities derived from the multivariate regression analysis as a percentage risk of positive CRM. Of the 1390 patients, 850 (61.2%) had no risk factors and were at low risk (risk < 5%) for CRM involvement, whereas 502 (36.1%) were at intermediate risk (risk 5–15%) and 48 (3.5%) of patients were at high risk (risk > 15%). Due to the limited number of patients with CRM positivity, there was one subgroup with combinations of risk factors where no events occurred, and subsequently, no predicted risk could be provided.

**Table 5:** The predicted risk (and cumulative score) for pCRM involvement according to the four independent risk factors

	cT1–3-stage		cT4-stage	
Sphincter saving procedure	No involved MRF on MRI	Involved MRF on MRI	No involved MRF on MRI	Involved MRF on MRI
Yes (LAR)	3.0% (0.0)	*6.8% (1.8)*	*7.1% (2.1)*	**15.0% (3.9)**
No (APR)	*6.6% (2.1)*	*13.5% (3.9)*	NA	**26.8% (6.0)**

Regular font indicates low (<5%) predicted risk of pCRM positivity; italic, intermediate (5–15%) predicted risk of pCRM positivity; bold, high (>15%) predicted risk of pCRM positivity. APR, abdominal perineal resection; CRM, circumferential resection margin; LAR, low anterior resection; MRF, mesorectal fascia.

## Discussion

In rectal cancer surgery, pathological involvement of the CRM remains a pivotal concern due to its substantial impact on potentially preventable locoregional recurrence rates^4,5^. This international multicentre retrospective cohort study assessing the incidence of positive CRM observed a notable 6.0% positive CRM rate, which can be considered an indirect marker of good surgical oncological performance. The study identified tumour- and procedure-related characteristics, specifically cT4 tumours, involved MRF on MRI and non-sphincter-saving surgery as predictive factors for CRM involvement. Remarkably, patient-related factors such as male sex, BMI, and previous abdominal surgery, known to pose greater technical challenges in conventional approaches, exhibited no discernible influence on CRM involvement rates following R-TME in this cohort.

This study demonstrates a 6.0% incidence of CRM involvement after R-TME in a multicentre international cohort. This finding indirectly indicates good surgical oncological performance, particularly considering this study excluded (pathological) complete responders. The relatively low incidence of CRM involvement may be attributed to the collective expertise of surgeons operating beyond their learning curve within highly specialized colorectal centres. Furthermore, using the robot-assisted surgical technique likely contributed to this favourable outcome. Current literature supports that the robotic technique enhances access to and provides superior visualization of the distal rectum. This facilitates precise oncologic dissection, elevating the overall quality of TME^20,21^. In a recently published randomized controlled trial, Feng *et al.*^22^ reported a higher rate of complete mesorectal resection of R-TME compared to L-TME, showcasing 559 (95.4%) of 586 patients achieving complete resection *versus* 537 (91.8%) of 585 patients in the L-TME group. Notably, the R-TME group exhibited a lower rate of positive CRM (22 (4.0%) of 547 *versus* 39 (7.2%) of 543; 95% c.i. −6.0 to −0.4; *P* = 0.023). However, it is important to note that the patients included in the REAL trial had lower BMIs than those in this cohort. The recently published multicentre prospective phase II VITRUVIANO trial reported a similar rate of positive CRM (4.6%) after R-TME compared to the results of the current study^23^. However, the VITRUVIANO trial only included patients with advanced rectal cancer, highlighting a narrower patient population compared to the broader cohort in the current analysis. The oncological superiority of the robot-assisted technique is further corroborated by a recent meta-analysis conducted by Khajeh *et al.*^24^. Their analysis included four studies and demonstrated a decreased risk of CRM involvement when comparing robot-assisted surgery with laparoscopic surgery, with an odds ratio of 1.56 (95% c.i. 1.11 to 2.20; *P* = 0.01). Although the current study emphasizes the oncological safety of R-TME, a direct head-to-head comparison between R-TME and L-TME was not conducted. As a result, this study does not establish the superiority of R-TME over L-TME in terms of positive CRM rates.

Unlike previous laparoscopic studies^25,26^, the current research uncovered no significant association between patient-related factors, such as male sex, obesity (BMI >30 kg/m^2^), previous abdominal surgery, and CRM outcomes following R-TME. This disparity in findings may be attributed to inherent limitations within laparoscopic surgery. Navigating the lower pelvis with rigid instrumentation during L-TME can pose formidable challenges even for seasoned colorectal surgeons. In stark contrast, robotic instruments' flexibility and enhanced precision likely contribute to improved manoeuvrability in the confined pelvic space, overcoming constraints encountered in conventional laparoscopy. Notably, a recent investigation by Roodbeen *et al.*^9^ assessing predictive factors for positive CRM after TaTME similarly concluded that patient-related factors held no predictive value for positive CRM. Instead, they reported tumour-related factors as the exclusive predictors for positive CRM. Consequently, the technical advantages inherent in robotic and transanal surgery, when performed by experienced surgeons, seem to mitigate the impact of challenging patient characteristics, thus endorsing the potential preference for these techniques within high-risk groups.

Tumour-related factors, encompassing cT4 tumours and involved MRF on MRI, emerged as independent predictors of positive CRM, aligning with established literature. Advanced preoperative T-stage emerged as an independent predictor of positive CRM, aligning with several studies^8,12,14^, including a comprehensive retrospective analysis of the United States National Cancer Database by Al-Sukhni *et al.*^8^. The correlation between involved MRF on MRI and CRM involvement has previously been described for TaTME by Roodbeen *et al.*^9^. The correlation between an advanced tumour stage on imaging and CRM positivity likely stems from direct tumour extension. However, it is noteworthy to acknowledge the nuanced nature of the literature. For example, a recent study by Hiranyakas *et al.*^27^ reported no significant association between T-stage and CRM positivity. Nevertheless, the exclusion of T4 lesions in their study may contribute to the discordant findings, given that T4 tumours may exert a more pronounced association with CRM positivity.

The current study identified non-sphincter-saving surgery as an independent predictor of positive CRM, aligning with prior research findings^8,28^. The challenges inherent in APR, marked by higher intraoperative perforation rates and increased CRM involvement, have been substantiated by various international studies^29,30^, underscoring the profound impact of the surgical approach on oncological outcomes. A multicentre RCT conducted by Guillou *et al.*^31^ in 27 centres across the UK reported a two-fold increased incidence of CRM involvement following APR compared to patients undergoing anterior resection (20 (23%) of 75 *versus* 17 (10%) of 193). This observation can be attributed to the fact that APR extends beyond the MRF onto the sphincters, and its predominantly abdominal approach places the critical area of resection, situated around the tumour itself, at the pelvic floor, where visualization and access are inherently limited. These challenges result in a heightened intraoperative perforation rate in low rectal cancer and a notable increase in CRM involvement^29^. Delving deeper into the nuances of APR, especially the extent of robotic-assisted intervention, could yield invaluable insights, albeit such data were not accessible in the present cohort. Subsequent investigations are encouraged to integrate this aspect into data collection protocols. Furthermore, despite accounting for various confounding factors in the multivariate model, selection bias among surgeons may still have existed, particularly with patients receiving an APR. In contrast to these findings, Hiranyakas *et al.*^27^ reported no significant association between APR and CRM positivity, a disparity potentially attributable to their study's modest sample size. Moreover, Roodbeen *et al.*^9^ similarly reported no significant association between APR and CRM positivity. However, it is imperative to acknowledge the differential indications for which TaTME and R-TME are employed, given that TaTME is infrequently utilized for APR procedures, resulting in a markedly lower rate of non-sphincter-saving surgery in the study by Roodbeen *et al.*^9^ compared to the present investigation (8.0% *versus* 23.4%). This variance may have contributed to the absence of an association between APR and CRM positivity in the study by Roodbeen *et al.*^9^.

The current study revealed no significant association between tumours within 1 cm from the ARJ and CRM positivity. This finding diverges from prior research by Roodbeen *et al.*^9^ and the Mercury II study^32^. The latter included patients diagnosed with low rectal cancer located ≤6 cm from the anal verge and reported significantly higher rates of CRM positivity in tumours located within <4 cm from the anal verge (OR 3.39; 95% c.i. to 1.3, 8.8; *P* = 0.012). The authors suggest that this association may be elucidated by the tapering of the mesorectum towards the anus, thereby reducing the range for obtaining clear margins. The incongruence between previous literature and the outcomes of the current study may be attributed to the potential capacity of the robot-assisted technique to mitigate challenges associated with the anatomical tapering of the mesorectum towards the anus. Conversely, it is conceivable that a significant correlation exists between tumours located within 1 cm from the ARJ and CRM positivity, especially considering the substantial overlap between patients with tumours near the ARJ and those undergoing APR, which itself emerged as a significant predictor for CRM positivity. Nonetheless, the current study is constrained by a relatively limited number of patients with tumours within 1 cm from the ARJ compared to the investigation by Roodbeen *et al.*^9^ (12.4% *versus* 19.7%). This disparity may have rendered the current study underpowered to demonstrate an association between tumours within 1 cm from the ARJ and CRM positivity. It is worth noting that our study included rectal cancers located within 15 cm of the ARJ. However, a recent study suggested that most tumours between 10 and 15 cm from the ARJ might be classified as sigmoid if the new sigmoidal take-off definition is used^33^. Unfortunately, this definition could not be applied in our cohort due to the inability to assess MRIs in some centres. As fewer than 100 patients had tumours between 10 and 15 cm from the ARJ, and among them, only a few exhibited positive CRM, we estimate that excluding these patients would not improve this study.

The present study uncovered no noteworthy association between EMVI identified on MRI and positive CRM, which diverges from the findings reported in prior literature. Previous studies consistently demonstrated a significantly worse prognosis and elevated rates of both local and systemic recurrence in patients with positive EMVI^34,35^. This incongruity could be elucidated by the evolving reporting standards for EMVI over the years and potential shortcomings in the documentation of EMVI during the initial years of our data set. Additionally, previous literature suggests particularly higher grades of EMVI correlate with positive CRM; however, regrettably, these specific data were not universally available across all data sets.

Statistical predictive risk models are crucial in anticipating oncological patient outcomes. The development of a predictive model for positive CRM in R-TME, as presented in this study, holds significant implications for both patients and surgeons. This risk-quantifying model informs decision-making during the consent process and empowers surgeons to identify high-risk patients, allowing for personalized interventions. The currently proposed risk prediction model aims to educate the patient and surgeon about the anticipated risk of CRM involvement and may be used only after extensive prospective validation to guide the appropriate operative strategy. This allows for strategic decisions such as selecting more experienced surgeons or opting for total neoadjuvant therapy rather than conventional chemoradiotherapy for patients at higher risk of positive CRM. This tailored approach can potentially enhance the curative efficacy of surgery, thereby advancing patient care and optimizing oncological outcomes.

This study is subject to several limitations. First, inherent to retrospective evaluations of surgical techniques is the potential for selection bias, whereby surgeons may preferentially select ‘complex’ cases for specific techniques. Despite efforts to mitigate this bias by consecutively including all patients from the international data set and applying exclusion criteria, the risk persists. Another limitation is the potential for institutional variation, as robot-assisted surgery performed across different centres was evaluated. Although only patients from centres where robot-assisted surgery was the standard approach for rectal cancer treatment were included, differences in postoperative care and surgical protocols could still influence the outcomes. Second, the retrospective study introduces potential inconsistencies due to a lack of standardized pathological assessment and reliance on local pathologists. Future research should prioritize a prospective design with standardized pathological assessments to ensure uniform reporting standards. Third, due to the low incidence of CRM involvement, only a limited number of potential predictors could be assessed. It is noteworthy, however, that the event rates were sufficient to evaluate all well-established a-priori risk factors for positive CRM, as documented in the current literature^7,8,12–17,25,27,36–43^. Subsequent studies should ensure sufficient power to investigate this outcome robustly. Fourth, the inclusion of only patients operated on by surgeons beyond their learning curves limits the universal applicability of the results. Furthermore, although our predictive model exhibited acceptable discrimination with an AUC of 0.71, its clinical utility may be somewhat constrained as it falls short of the typically significant threshold (>0.80)^44^. Nonetheless, these findings provide a foundation for further refinement of predictive modelling within surgical practice. Lastly, internal validation was conducted for the predictive model in the present study. However, future investigations should extend to external validation to enhance the model's generalizability before definitive conclusions are drawn. Recognizing and addressing these limitations will contribute to the refinement and applicability of future research in this domain.

## Collaborators

All collaborators of the EUREKA study group. For the Netherlands, the MIRECA study group: G.J.D. van Acker, T.S. Aukema, H.J. Belgers, F.H. Beverdam, J.G. Bloemen, K. Bosscha, S.O. Breukink, P.P.L.O. Coene, R.M.P.H. Crolla, P. van Duijvendijk, E.B. van Duyn, I.F. Faneyte, S.A.F. Fransen, A.A.W. van Geloven, M.F. Gerhards, W.M.U. van Grevenstein, K. Havenga, I.H.J.T. de Hingh, C. Hoff, G. Kats, J.W.A. Leijtens, M.F. Lutke Holzik, J. Melenhorst, M.M. Poelman, A. Pronk, A.H.W. Schiphorst, J.M.J. Schreinemakers, C. Sietses, A.B. Smits, I. Somers, E.J. Spillenaar Bilgen, H.B.A.C. Stockmann, A.K. Talsma, P.J. Tanis, J. Tuynman, E.G.G. Verdaasdonk, F.A.R.M. Warmerdam, H.L. van Westreenen, D.D.E. Zimmerman. For France, the ICM study group: Quentin Denost, Christina Fleming, Bordeaux Colorectal Institute, Clinique Tivoli, Bordeaux, France. Philipe Rouanet, Nabila Bouazza, Aurore Moussion, Surgery and Clinical Research Department, Montpellier Cancer Institute (ICM), Univ. Montpellier, Montpellier, France. Eddy Cotte, Department of Digestive and Oncological Surgery, Lyon University Hospital, Lyon-Sud Hospital, Pierre-Bénite, France. Anne Dubois, Department of Colorectal Surgery, Chu Estaing, Clermont-Ferrand, France. Eric Rullier, Department of Digestive Surgery, Colorectal Unit, Haut-Lévêque Hospital, Bordeaux University Hospital, Pessac, France. For the United Kingdom, the Portsmouth Colorectal group: J. Conti, G. David, D. O'Leary, A. Przedlacka, M. Rabie, J. Richardson, F. Sagias, P. Sykes, Portsmouth Hospitals University NHS Trust, Department of Colorectal Surgery, Portsmouth, UK.

## Supplementary Material

zraf027_Supplementary_Data

## Data Availability

Anonymized data could be made available from the corresponding author following reasonable request. In compliance with what has been agreed to in the consortium agreement and informed consent, pseudonymized data will be made accessible to other researchers through dataverse.nl (with restricted access) if they comply with Dutch legislation and comply with any restrictions that the ethics committee might impose on the reuse. To do so, researchers would have to contact the EUREKA Steering Committee.
