# Supplementary material for: Predictors and risk model for positive circumferential resection margin after robot-assisted total mesorectal excision: retrospective cohort study
Source: BJS Open. 2025 May 22;9(3):zraf027. doi: 10.1093/bjsopen/zraf027 (PMC12097205; doi:10.1093/bjsopen/zraf027)
Supplement: zraf027_Supplementary_Data [file zraf027_supplementary_data.docx]

**Predictors and Risk Model for Positive Circumferential Resection Margin after Robot-Assisted Total Mesorectal Excision: Retrospective cohort study**

Ritch T.J. Geitenbeek, MD1,2, Thijs A. Burghgraef, MD, PhD1,2, Rauand Duhoky, MD3, Christina A. Fleming, MD, PhD4, Aurore Moussion5, Nabila Bouazza5, Eddy Cotte6, Anne Dubois7, Eric Rullier8, Quentin Denost, MD, PhD4, Philippe Rouanet, MD, Phd9, Jim Khan, MD, PhD3, Roel Hompes, MD, PhD10,11*, Esther C. J. Consten, MD, PhD1,2*, on behalf of the EUREKA study group

^1^University of Groningen, University Medical Centre Groningen, Department of Surgery, Groningen, the Netherlands

^2^Meander Medical Centre, Department of Surgery, Amersfoort, the Netherlands

^3^Portsmouth Hospitals University NHS Trust and the University of Portsmouth, UK

^4^Bordeaux Colorectal Institute, Clinique Tivoli, Bordeaux, France

^5^Clinical Research Department, Montpellier Cancer Institute (ICM), Univ. Montpellier, Montpellier, France

^6^Department of Digestive and Oncological Surgery, Lyon University Hospital, Lyon-Sud Hospital, Pierre-Bénite, France

^7^Department of Colorectal Surgery, Chu Estaing, Clermont-Ferrand, France

^8^Department of Digestive Surgery, Colorectal Unit, Haut-Lévêque Hospital, Bordeaux University Hospital, Pessac, France

^9^Surgery Department, Montpellier Cancer Institute (ICM), Univ. Montpellier, Montpellier, France

^10^University of Amsterdam, University Medical Centre Amsterdam, Department of Surgery, Amsterdam, the Netherlands

^11^Amsterdam Cancer Centre, Department of Surgery, Amsterdam, the Netherlands

^*^Both authors contributed equally and shared the last authorship

**Esther C.J. Consten.** University Medical Centre Groningen, Department of Surgery. **0000-0002-9447-8181**.

**Supplementary Materials - Index**

| **Supplementary Methods** |  |
| --- | --- |
| Supplemental File 1. | *pag. 3* |
| Detail | *pag. Y* |
| **Supplementary Results** |  |
| Detail | *pag. X* |
| Detail | *pag. Y* |
| **Supplementary Appendixes** |  |
| Detail | *pag. X* |
| Detail | *pag. Y* |
| **Supplementary Figures and Tables** |  |
| Supplemental Figure 1. | *pag. 6* |
| Supplemental Figure 2. | *pag. 7* |
| Supplemental Figure 3. | *pag. 8* |
| **References** | *pag. Z* |
|  | *pag. 9* |

**Supplementary Methods**

**Supplemental File 1.** Definitions of extracted data and outcome parameters.

Rectal carcinoma was defined as tumors situated within 15 centimeters of the ARJ. The distance to the ARJ was measured from the proximal border of the tumor to the ARJ using magnetic resonance imaging (MRI). The presence of extramural venous invasion (EMVI) on baseline MRI was defined as intravenous tumor extension beyond the rectal wall. The presence of an involved mesorectal fascia (MRF) on baseline MRI was defined as a tumor within 1 mm of the MRF. The MRI response to NAT was categorized as either a 'response' or 'no response,' accounting for differences in response grading among the participating countries in the EUREKA collaboration. Surgical procedures were grouped into three categories: low anterior resection (LAR) with anastomosis, LAR without anastomosis (ultra-low Hartmann procedure), and abdominoperineal resection (APR). APR was defined as a complete rectal resection with intersphincteric or extrasphincteric dissection and end colostomy. If only a mucosectomy was performed in combination with excision of the anal canal, this was recorded as an intersphincteric APR. Both restorative LAR and non-restorative LAR were considered sphincter-saving surgery, whilst APR was considered non-sphincter-saving surgery. Mucinous adenocarcinoma was defined as a tumor in which more than 50% of the tissue displayed a mucinous pattern upon histological examination. The quality of the TME specimen was evaluated based on the principles outlined by Nagtegaal et al.

**Supplementary Results**

**Supplementary Appendixes**

**Supplementary Figures and Tables**

**Supplemental Figure 1.** Flowchart of patient inclusion from the EUREKA cohort.


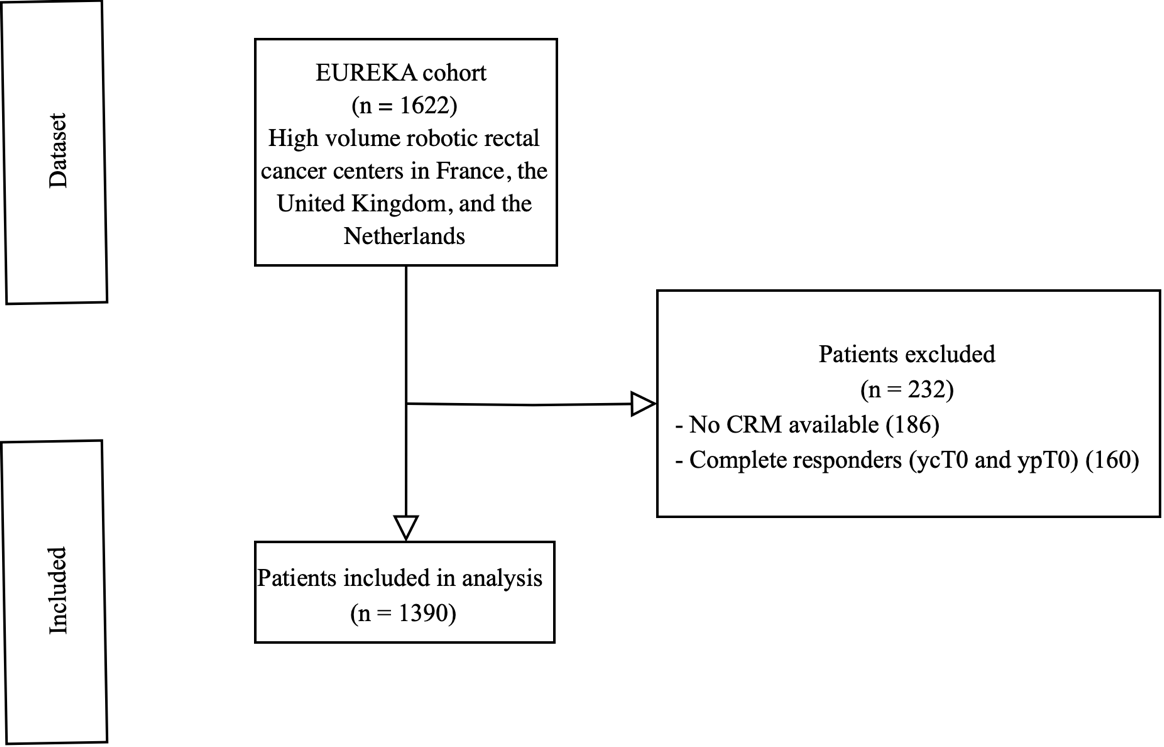


*Abbreviations: CRM,* circumferential resection margin; *EUREKA,* Expert Dutch, French, and UK Robotic Rectal Cancer Centers; *n,* total number of patients.

**Supplemental Figure 2.** Discrimination of predictive model.


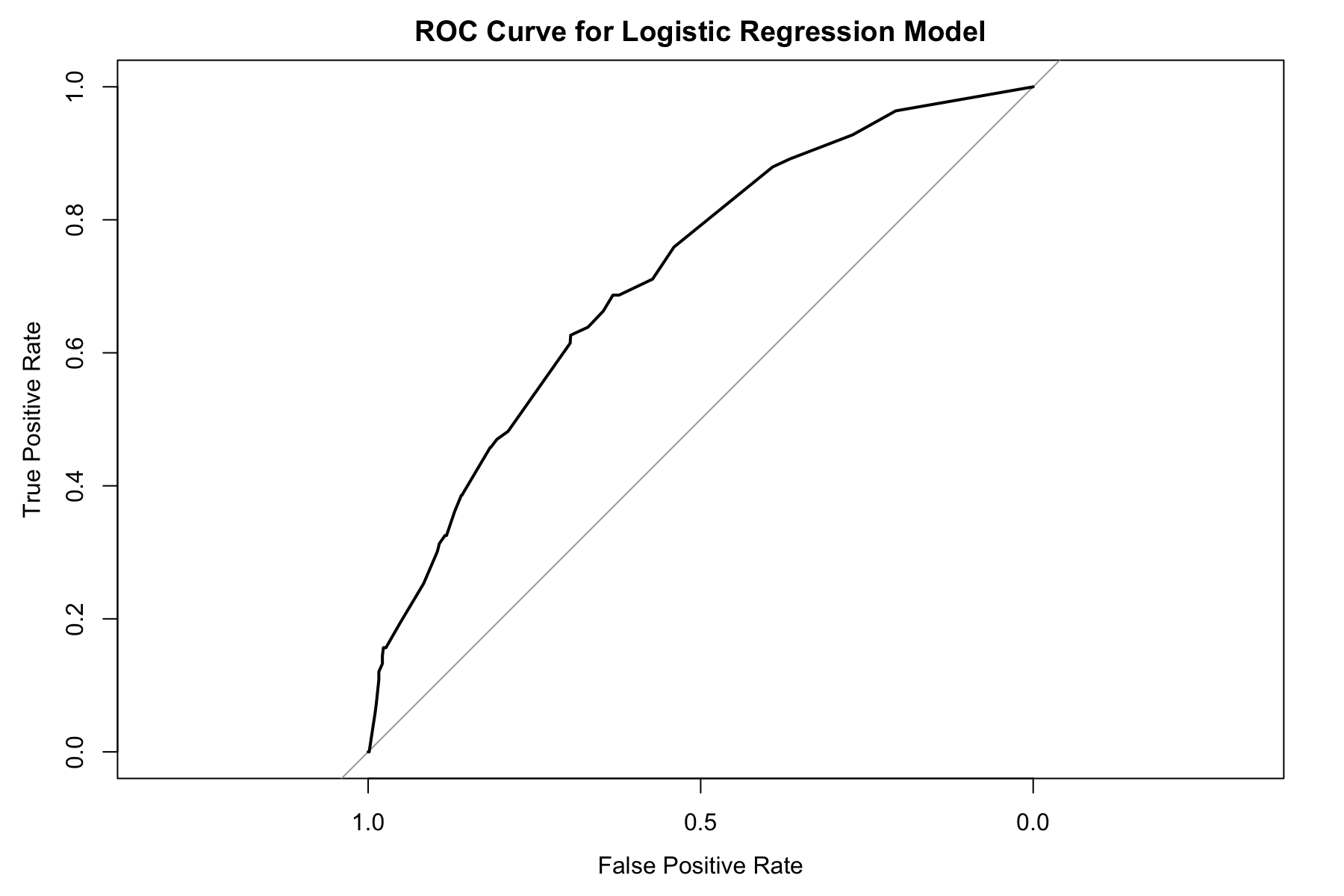


Apparent c-statistic AUC 0.716 (CI: 0.660-0.716). After correcting for optimism, the c-statistic (corrected AUC) was 0.712.

*Abbreviations: AUC,* area under the received operating curve; *CRM,* circumferential resection margin; *ROC,* receiver operating curve.

**Supplemental Figure 3.** Comparison of observed and model-predicted risk of positive CRM.


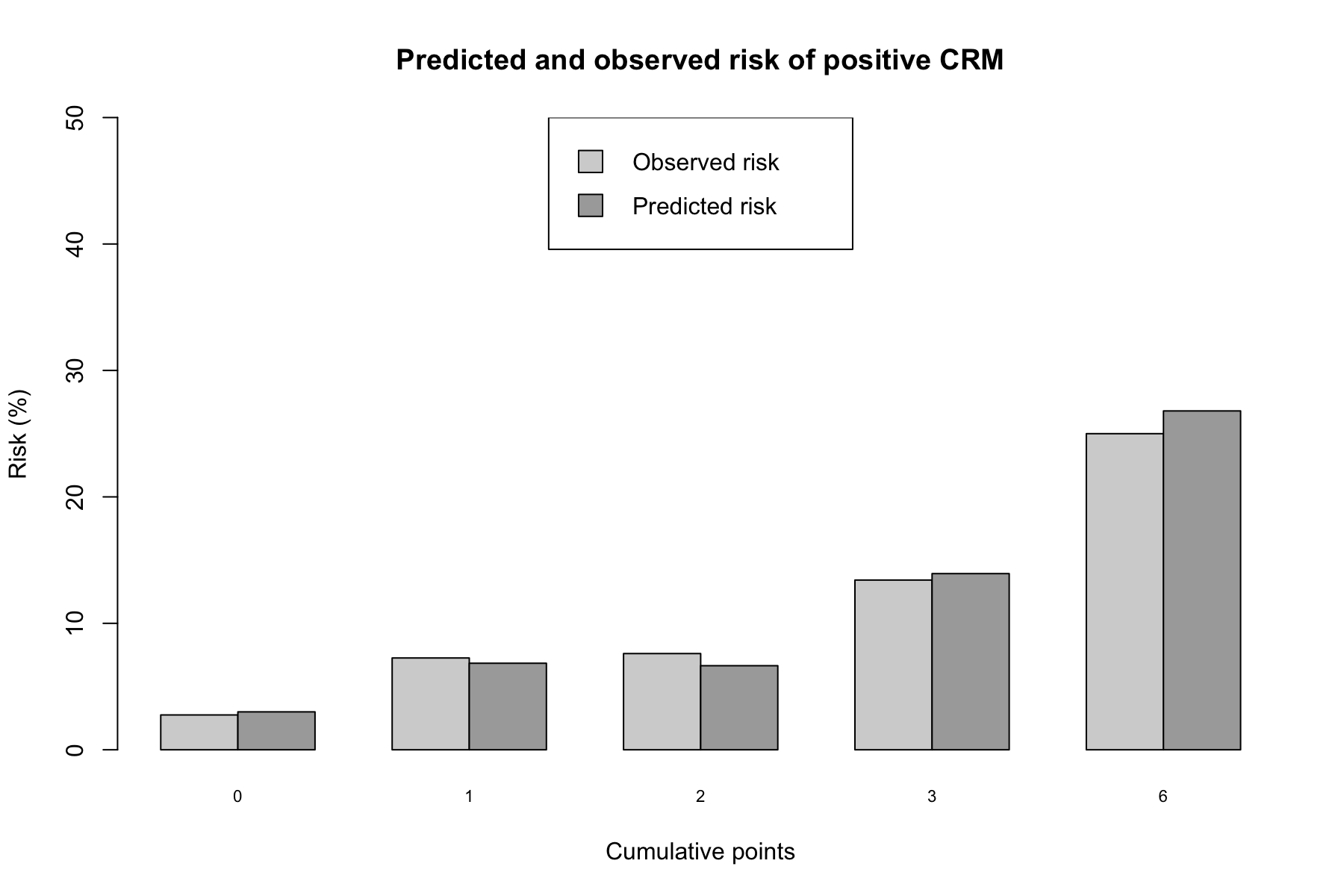


*Abbreviations: CRM,* circumferential resection margin.

**References**

1 Heald RJ, Ryall RDH. Recurrence and survival after total mesorectal excision for rectal cancer. *The Lancet*. Elsevier; 1986 Jun 28; **327**: 1479–1482.

2 Chen K, Cao G, Chen B, Wang M, Xu X, Cai W, *et al.* Laparoscopic versus open surgery for rectal cancer: A meta-analysis of classic randomized controlled trials and high-quality Nonrandomized Studies in the last 5 years. *International Journal of Surgery*. M. Xiong, Department of Gastrointestinal Surgery, The First Affiliated Hospital of Anhui Medical University, Hefei, Anhui, China, England; 2017 Mar; **39**: 1–10.

3 Jayne D, Pigazzi A, Marshall H, Croft J, Corrigan N, Copeland J, *et al.* Effect of Robotic-Assisted vs Conventional Laparoscopic Surgery on Risk of  Conversion to Open Laparotomy Among Patients Undergoing Resection for Rectal Cancer: The ROLARR Randomized Clinical Trial. *JAMA*. 2017 Oct; **318**: 1569–1580.

4 Nagtegaal ID, Quirke P. What is the role for the circumferential margin in the modern treatment of rectal cancer? *Journal of Clinical Oncology*. I.D. Nagtegaal, Department of Pathology 824, University Medical Centre Nijmegen, PO Box 9101, 6500 HB, Nijmegen, Netherlands: American Society of Clinical Oncology; 2008 Jan 10; **26**: 303–312.

5 Quirke P, Dixon MF, Durdey P, Williams NS. Local recurrence of rectal adenocarcinoma due to inadequate surgical resection. Histopathological Study of Lateral Tumour Spread and Surgical Excision. *The Lancet*. Lancet; 1986 Nov 1; **328**: 996–999.

6 Kang BM, Park YK, Park SJ, Lee KY, Kim CW, Lee SH. Does circumferential tumour location affect the circumferential resection margin status in mid and low rectal cancer? *Asian J Surg*. Elsevier (Singapore) Pte Ltd; 2018 May 1; **41**: 257–263.

7 Homan J, Bökkerink GM, Aarts MJ, Lemmens VE, van Lijnschoten G, Rutten HJ, *et al.* Variation in circumferential resection margin: Reporting and involvement in the South-Netherlands. *European Journal of Surgical Oncology*. J. Homan, Department of Surgery, Radboud University Medical Centre Nijmegen, Geert Grooteplein-Zuid 10, Nijmegen, Netherlands, England; 2015 Nov; **41**: 1485–1492.

8 Al-Sukhni E, Attwood K, Gabriel E, Nurkin SJ. Predictors of circumferential resection margin involvement in surgically resected rectal cancer: A retrospective review of 23,464 patients in the US National Cancer Database. *Int J Surg* [Internet]. NIH Public Access; 2016 Apr 1 [cited 2024 Oct 4]; **28**: 112. Available from: /pmc/articles/PMC5295134/

9 Roodbeen SX, De Lacy FB, Van Dieren S, Penna M, Ris F, Moran B, *et al.* Predictive Factors and Risk Model for Positive Circumferential Resection Margin Rate after Transanal Total Mesorectal Excision in 2653 Patients with Rectal Cancer. *Ann Surg*. R. Hompes, Department of Surgery, Amsterdam University Medical Centres, University of Amsterdam, Cancer Centre Amsterdam, Amsterdam, Netherlands: Lippincott Williams and Wilkins; 2019; **21**: 11.

10 Fleming CA, Duhoky R, Geitenbeek RTJ, Moussion A, Bouazza N, Khan J, *et al.* Multicentre cohort study evaluating clinical, oncological and functional outcomes following robotic rectal cancer surgery—the EUREKA collaborative: trial protocol. *BJS Open* [Internet]. Oxford University Press; 2024 Mar 1 [cited 2024 Apr 12]; **8**. Available from: /pmc/articles/PMC10996923/

11 von Elm E, Altman DG, Egger M, Pocock SJ, Gøtzsche PC, Vandenbroucke JP. The Strengthening the Reporting of Observational Studies in Epidemiology (STROBE) statement: guidelines for reporting observational studies. *J Clin Epidemiol*. Elsevier; 2008 Apr 1; **61**: 344–349.

12 Warrier SK, Kong JC, Guerra GR, Chittleborough TJ, Naik A, Ramsay RG, *et al.* Risk factors associated with circumferential resection margin positivity in rectal cancer: A binational registry study. *Dis Colon Rectum*. S.K. Warrier, Division of Cancer Surgery, Peter MacCallum Cancer Centre, Melbourne, VIC, Australia: Lippincott Williams and Wilkins; 2018; **61**: 433–440.

13 van Leersum N, Martijnse I, den Dulk M, Kolfschoten N, Le Cessie S, van de Velde C, *et al.* Differences in circumferential resection margin involvement after abdominoperineal excision and low anterior resection no longer significant. *Ann Surg*. H.J. Rutten, Department Surgery, Catharina Hospital, Michelangelolaan 2, 5623 EJ Eindhoven, Netherlands, United States; 2014 Jun; **259**: 1150–1155.

14 Rickles AS, Dietz DW, Chang GJ, Wexner SD, Berho ME, Remzi FH, *et al.* High rate of positive circumferential resection margins following rectal cancer surgery a call to action. *Ann Surg*. J.R.T. Monson, Surgical Health Outcomes and Research Enterprise, Department of Surgery, University of Rochester Medical Centre, Box SURG, 601 Elmwood Avenue, Rochester, NY, United States; 2015 Dec; **262**: 891–898.

15 Son I, Kim Y, Lee K, Kang S, Kim D, Shin E, *et al.* Oncologic relevance of magnetic resonance imaging–detected threatened mesorectal fascia for patients with mid or low rectal cancer: A longitudinal analysis before and after long-course, concurrent chemoradiotherapy. *Surgery (United States)*. S.-B. Kang, Department of Surgery, Seoul National University Bundang Hospital, 166 Gumi-ro, Bundang-gu, Seongnam, South Korea; 2017; **162**: 152–163.

16 Algebally AM, Mohey N, Szmigielski W, Yousef RRH, Kohla S. The value of high-resolution MRI technique in patients with rectal carcinoma: Pre-operative assessment of mesorectal fascia involvement, circumferential resection margin and local staging. *Pol J Radiol*. W. Szmigielski, Department of Clinical Imaging, National Centre for Cancer Care and Research, Hamad Medical Corporation, P.O. Box 3050, Doha, Qatar; 2015; **80**: 115–121.

17 Zhang G, Cai Y-Z, Xu G-H, G. Z, Y.-Z. C, G.-H. X. Diagnostic Accuracy of MRI for Assessment of T Category and Circumferential Resection Margin Involvement in Patients with Rectal Cancer: A Meta-Analysis. *Dis Colon Rectum*. G.-H. Xu, Department of Radiology, Sichuan Cancer Hospital, No. 55, Section 4, South Renmin Road, Chengdu, Sichuan, China, United States; 2016 Aug; **59**: 789–799.

18 Steyerberg EW, Harrell FE, Borsboom GJJM, Eijkemans MJC, Vergouwe Y, Habbema JDF. Internal validation of predictive models: efficiency of some procedures for logistic regression analysis. *J Clin Epidemiol*. J Clin Epidemiol; 2001; **54**: 774–781.

19 Smith GCS, Seaman SR, Wood AM, Royston P, White IR. Correcting for optimistic prediction in small data sets. *Am J Epidemiol*. Am J Epidemiol; 2014 Aug 1; **180**: 318–324.

20 Nagtegaal ID, Quirke P. What is the role for the circumferential margin in the modern treatment of rectal cancer? *J Clin Oncol*. J Clin Oncol; 2008 Jan 10; **26**: 303–312.

21 Quirke P, Dixon MF, Durdey P, Williams NS. Local recurrence of rectal adenocarcinoma due to inadequate surgical resection. Histopathological study of lateral tumour spread and surgical excision. *Lancet*. Lancet; 1986 Nov 1; **2**: 996–999.

22 Veltcamp Helbach M, Koedam TWA, Knol JJ, Velthuis S, Bonjer HJ, Tuynman JB, *et al.* Quality of life after rectal cancer surgery: differences between laparoscopic and transanal total mesorectal excision. *Surg Endosc*. 2019; **33**: 79–87.

23 Nasir M, Panteleimonitis S, Ahmed J, Abbas H, Parvaiz A. Learning Curves in Robotic Rectal Cancer Surgery : A literature Review. *Journal of minimally invasive surgical sciences*. 2016; **5**.

24 Feng Q, Yuan W, Li T, Tang B, Jia B, Zhou Y, *et al.* Robotic versus laparoscopic surgery for middle and low rectal cancer (REAL): short-term outcomes of a multicentre randomised controlled trial. *Lancet Gastroenterology ad Hepatology*. 2022; **7**: 991–1004.

25 Hamabe A, Takemasa I, Kotake M, Nakano D, Hasegawa S, Shiomi A, *et al.* Feasibility of robotic-assisted surgery in advanced rectal cancer: a multicentre prospective phase II study (VITRUVIANO trial). *BJS Open* [Internet]. BJS Open; 2024 Jun 1 [cited 2024 Oct 2]; **8**. Available from: https://pubmed.ncbi.nlm.nih.gov/38913419/

26 Khajeh E, Aminizadeh E, Dooghaie Moghadam A, Nikbakhsh R, Goncalves G, Carvalho C, *et al.* Outcomes of Robot-Assisted Surgery in Rectal Cancer Compared with Open and Laparoscopic Surgery. *Cancers (Basel)*. MDPI; 2023 Feb 1; **15**.

27 Russell MC, You YN, Hu CY, Cormier JN, Feig BW, Skibber JM, *et al.* A novel risk-adjusted nomogram for rectal cancer surgery outcomes. *JAMA Surg*. G.J. Chang, Department of Surgical Oncology, University of Texas MD Anderson Cancer Centre, 1400 Pressler St, FCT 17.6000, Houston, TX 77030, United States: NIH Public Access; 2013 Aug; **148**: 769–777.

28 Hasegawa S, Takahashi R, Hida K, Kawada K, Sakai Y. Transanal total mesorectal excision for rectal cancer. Surg Today. Springer Tokyo; 2016. p. 641–653.

29 Hiranyakas A, da Silva G, Wexner SD, Ho Y-H, Allende D, Berho M, *et al.* Factors influencing circumferential resection margin in rectal cancer. *Colorectal Disease*. M. Berho, Department of Anatomic Pathology, 2950 Cleveland Clinic Blvd, Weston, FL 33331, United States: John Wiley & Sons, Ltd; 2013 Mar 1; **15**: 298–303.

30 Rullier A, Gourgou-Bourgade S, Jarlier M, Bibeau F, Chassagne-Clément C, Hennequin C, *et al.* Predictive factors of positive circumferential resection margin after radiochemotherapy for rectal cancer: The French randomised trial ACCORD12/0405 PRODIGE 2. *Eur J Cancer*. A. Rullier, Department of Pathology, CHU Bordeaux, Place Amélie Raba-Léon, 33076 Bordeaux Cedex, France, England; 2013 Jan; **49**: 82–89.

31 Nagtegaal ID, Van De Velde CJH, Marijnen CAM, Van Krieken JHJM, Quirke P. Low rectal cancer: a call for a change of approach in abdominoperineal resection. *J Clin Oncol*. J Clin Oncol; 2005; **23**: 9257–9264.

32 Leonard D, Penninckx F, Fieuws S, Jouret-Mourin A, Sempoux C, Jehaes C, *et al.* Factors predicting the quality of total mesorectal excision for rectal cancer. *Ann Surg*. F. Penninckx, Department of Abdominal Surgery, University Clinic Gasthuisberg, Herestraat 49, 3000-Leuven, Belgium, United States; 2010 Dec; **252**: 982–988.

33 Guillou PJ, Quirke P, Thorpe H, Walker J, Jayne DG, Smith AMH, *et al.* Short-term endpoints of conventional versus laparoscopic-assisted surgery in patients with colorectal cancer (MRC CLASICC trial): multicentre, randomised controlled trial. *Lancet*. Lancet; 2005 May 14; **365**: 1718–1726.

34 Battersby NJ, How P, Moran B, Stelzner S, West NP, Branagan G, *et al.* Prospective validation of a low rectal cancer magnetic resonance imaging staging system and development of a local recurrence risk stratification model: The mercury II study. *Ann Surg*. G. Brown, Department of Radiology, Royal Marsden Hospital NHS Trust, Downs Rd, Sutton, United Kingdom, United States; 2016 Apr; **263**: 751–760.

35 D’Souza N, de Neree tot Babberich MPM, d’Hoore A, Tiret E, Xynos E, Beets-Tan RGH, *et al.* Definition of the Rectum: An International, Expert-based Delphi Consensus. *Ann Surg* [Internet]. Ann Surg; 2019 Dec 1 [cited 2024 Feb 1]; **270**: 955–959. Available from: https://pubmed.ncbi.nlm.nih.gov/30973385/

36 Bugg WG, Andreou AK, Biswas D, Toms AP, Williams SM. The prognostic significance of MRI-detected extramural venous invasion in rectal carcinoma. *Clin Radiol*. Clin Radiol; 2014; **69**: 619–623.

37 Chand M, Siddiqui MRS, Swift I, Brown G. Systematic review of prognostic importance of extramural venous invasion in rectal cancer. *World J Gastroenterol*. Baishideng Publishing Group Inc; 2016 Jan 28; **22**: 1721–1726.

38 Oh SJ, Shin JY. Risk factors of circumferential resection margin involvement in the patients with extraperitoneal rectal cancer. *J Korean Surg Soc*. 2012 Mar; **82**: 165–171.

39 Tilney HS, Tekkis PP, Sains PS, Constantinides VA, Heriot AG, H.S. T, *et al.* Factors affecting circumferential resection margin involvement after rectal cancer excision. *Dis Colon Rectum*. P.P. Tekkis, Department of Biosurgery and Surgical Technology, Imperial College London, St. Mary’s Hospital, Praed Street, London, W2 1NY, United Kingdom, United States; 2007 Jan; **50**: 29–36.

40 Simpson GS, Eardley N, McNicol F, Healey P, Hughes M, Rooney PS. Circumferential resection margin (CRM) positivity after MRI assessment and adjuvant treatment in 189 patients undergoing rectal cancer resection. *Int J Colorectal Dis*. G.S. Simpson, Department of General Surgery, Royal Liverpool University Hospital, Liverpool, United Kingdom, Germany; 2014 May; **29**: 585–590.

41 Jiang J, Dai Y, Zhang X, Li C, Jin Z, Bi D, *et al.* Accuracy of preoperative magnetic resonance imaging in prediction of pathological stage and circumferential resection margin in rectal cancer. *National Medical Journal of China*. Y. Dai, Department of General Surgery, Qilu Hospital of Shandong University, Jinan 250012, China, China; 2006 Apr; **86**: 961–964.

42 Kim S, Lee J, Park H, Eun H, Han J, Choi B. Accuracy of MRI for Predicting the Circumferential Resection Margin, Mesorectal Fascia Invasion, and Tumour Response to Neoadjuvant Chemoradiotherapy for Locally Advanced Rectal Cancer. *Journal of Magnetic Resonance Imaging*. J. M. Lee, Department of Radiology, Seoul National University, College of Medicine, Yongon-dong 28, Chongno-gu, 110-744 Seoul, South Korea; 2009; **29**: 1093–1101.

43 Iannicelli E, Di Renzo S, Ferri M, Pilozzi E, Di Girolamo M, Sapori A, *et al.* Accuracy of high-resolution MRI with lumen distention in rectal cancer staging and circumferential margin involvement prediction. *Korean J Radiol*. E. Iannicelli, Radiology Institute Departments of Surgical and Medical Sciences and Translational Medicine, University of Rome, Sapienza, Sant’Andrea Hospital, Via di Grottarossa 1035, Rome 00189, Italy; 2014; **15**: 37–44.

44 Videhult P, Smedh K, Lundin P, Kraaz W, P. V, K. S, *et al.* Magnetic resonance imaging for preoperative staging of rectal cancer in clinical practice: High accuracy in predicting circumferential margin with clinical benefit. *Colorectal Disease*. K. Smedh, Colorectal Unit, Department of Surgery, Central Hospital, 721 89 Västerås, Sweden, England; 2007 Jun; **9**: 412–419.

45 Lahaye MJ, Engelen SME, Nelemans PJ, Beets GL, van de Velde CJH, van Engelshoven JMA, *et al.* Imaging for predicting the risk factors--the circumferential resection margin and  nodal disease--of local recurrence in rectal cancer: a meta-analysis. *Semin Ultrasound CT MR*. United States; 2005 Aug; **26**: 259–268.

46 de Hond AAH, Steyerberg EW, van Calster B. Interpreting area under the receiver operating characteristic curve. *Lancet Digit Health* [Internet]. Lancet Digit Health; 2022 Dec 1 [cited 2024 May 1]; **4**: e853–e855. Available from: https://pubmed.ncbi.nlm.nih.gov/36270955/
